# Diagnostic performance of non-invasive, stool-based molecular assays in patients with paucibacillary tuberculosis

**DOI:** 10.1038/s41598-020-63901-z

**Published:** 2020-04-28

**Authors:** Mohita Gaur, Anoop Singh, Vishal Sharma, Gayatri Tandon, Ankur Bothra, Aarushi Vasudeva, Shreeya Kedia, Ashwani Khanna, Vishal Khanna, Sheelu Lohiya, Mandira Varma-Basil, Anil Chaudhry, Richa Misra, Yogendra Singh

**Affiliations:** 1 Department of Zoology, University of Delhi, Delhi, India; 2 State TB Officer & In-Charge, Chest Clinic, Lok Nayak Hospital, Delhi, India; 3 Chest Clinic, Lok Nayak Hospital, Delhi, India; 4 Vallabhbhai Patel Chest Institute, University of Delhi, Delhi, India; 5 Rajan Babu Institute of Pulmonary Medicine and Tuberculosis, Kingsway Camp, Delhi, India; 6 Department of Zoology, Sri Venkateswara College, University of Delhi, Delhi, India

**Keywords:** Bacterial infection, Tuberculosis

## Abstract

Timely diagnosis of paucibacillary tuberculosis (TB) which includes smear-negative pulmonary TB (PTB) and extra-pulmonary TB (EPTB) remains a challenge. This study was performed to assess the diagnostic utility of stool as a specimen of choice for detection of mycobacterial DNA in paucibacillary TB patients in a TB-endemic setting. Stool samples were collected from 246 subjects including 129 TB patients (62 PTB and 67 EPTB) recruited at TB hospital in Delhi, India. Diagnostic efficacy of stool IS6110 PCR (*n* = 228) was measured, using microbiologically/clinically confirmed TB as the reference standard. The clinical sensitivity of stool PCR was 97.22% (95% confidence interval (CI), 85.47-99.93) for detection of *Mycobacterium tuberculosis* in stool samples of smear-positive PTB patients and 76.92% (CI, 56.35–91.03) in samples from smear-negative PTB patients. Overall sensitivity of PCR for EPTB was 68.66% (CI, 56.16–79.44), with the highest sensitivity for stool samples from patients with lymph node TB (73.5%), followed by abdominal TB (66.7%) and pleural effusion (56.3%). Stool PCR presented a specificity of 95.12%. The receiver operating characteristic curve also indicated the diagnostic utility of stool PCR in TB detection (AUC: 0.882). The performance characteristic of the molecular assay suggests that stool DNA testing has clinical value in detection of TB.

## Introduction

Tuberculosis (TB), a global health problem, is caused by *Mycobacterium tuberculosis* (*Mtb*) and has two ways of clinical manifestation. Apart from the common pulmonary TB (PTB) manifestation, extrapulmonary TB (EPTB) is identified as a major concern and contributes significantly to the disease burden worldwide. According to the World Health Organization (WHO), 10 million people fell ill with TB in 2018^1^. Although new strategies for diagnosis and treatment over the past decade have led to decline in death rate, it remains inadequate to reach the milestone of the WHO “End-TB” goal^1^. Apart from the diagnostic and treatment difficulties faced in the eradication of PTB, timely diagnosis and treatment of EPTB is considered a bigger challenge. This is due to the diverse, non-specific and paucibacillary presentation of EPTB at remote body sites and requirement of a longer treatment regimen^2^. In 2018, an increase in EPTB incidence was reported in India^3^, which is also considered as a TB high-burden country by the WHO. A probable explanation for this rise can be in part due to access to better diagnostic tools that highlight the fact that EPTB burden may have been underrepresented over the years. Thus, accurate and rapid diagnostic tests for PTB and EPTB are key to limit the spread of the epidemic.

**Figure 1:**
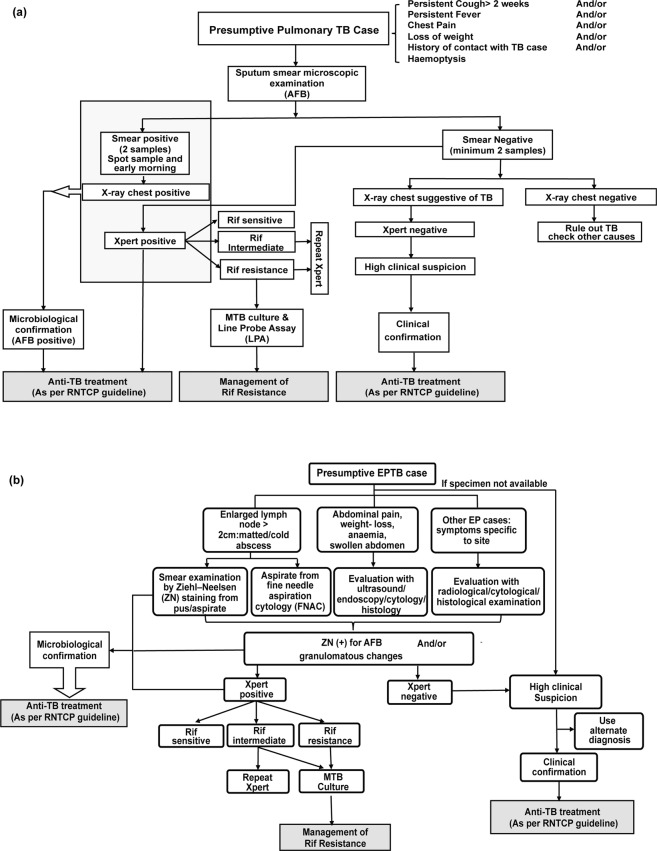
Diagnostic algorithm for Tuberculosis as per Revised National TB Control Program (RNTCP) guidelines: (Information source: Central TB Division, Ministry of Health and Family Welfare, Government of India). (**a**) Recommended diagnostic algorithm for pulmonary TB. (**b**) Recommended diagnostic algorithm for extra-pulmonary TB Abbreviations: TB, tuberculosis; AFB, acid fast bacilli; Rif, rifampicin.

In India, diagnosis of TB is done by the Revised National Tuberculosis Control Program guidelines that follow the WHO recommendations (Fig. 1). At present, although the diagnosis of PTB by sputum-smear direct microscopy is considerably established, the diagnosis of smear-negative PTB and EPTB poses serious challenges due to paucibacillary nature of the specimen. Diagnosis of EPTB usually involves sample procurement from the affected body site through invasive and painful procedures that requires clinical expertise. Advent of Xpert MTB/RIF (Xpert), an automated molecular detection system that simultaneously detects TB and resistance to rifampicin, proved to be a breakthrough in TB diagnosis^4^. However, the difficulty associated with specimen acquisition in paucibacillary cases persists. So, molecular tests with alternate clinical samples, that are easy to obtain, safer, and give uniform results, are warranted. Assays such as tuberculin skin test and T-cell based interferon-gamma release assay find limited utility in TB diagnosis in endemic countries like India. Culture identification also has limited role in routine diagnosis in India and is recommended for screening of drug-resistance^5,6^. Many recent studies have tested efficacy of stool in molecular diagnosis of pediatric PTB, while scarce reports are available on adult stool testing for detection of PTB from other countries^7–16^.

The present study aims to evaluate the effectiveness of stool sample for *Mtb* detection in varied TB cases by polymerase chain reaction (PCR) amplification of insertion element IS6110 in 228 subjects. Also, in a pilot run of 36 samples, stool Xpert testing was done for patients with PTB and EPTB to check for adaptability of stool testing in a tertiary healthcare setting.

## Results

**Figure 2:**
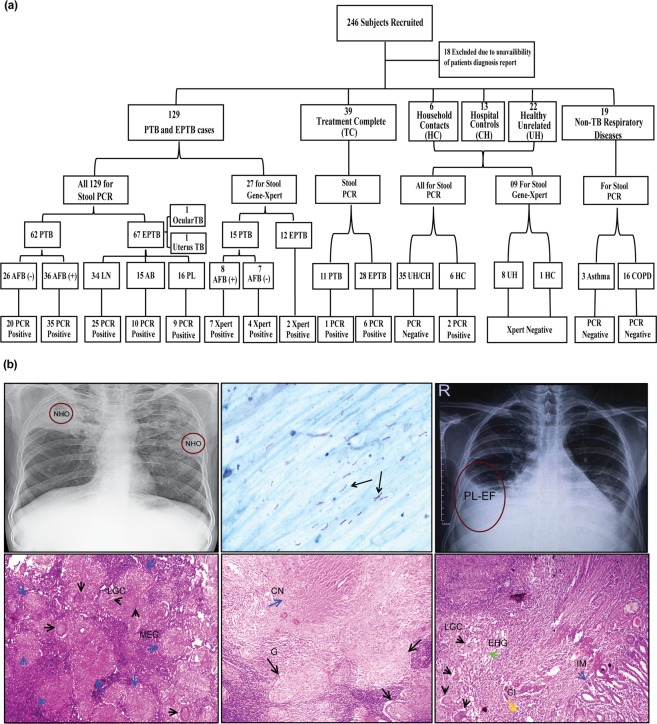
Study profile and representative diagnostic findings of TB patients. (**a**) Flowchart depicting the categorization of the study subjects. (**b**) Panel showing representative diagnostic images of TB patients, *upper left*: radiological examination showing non-homogenous opacity (NHO), marked by red circles; *upper middle*: acid fast stained tubercle bacilli, marked by black arrows; *upper right*: obliteration of right costophrenic angle suggestive of pleural effusion; *lower left*: tubercular lymphadenitis, photomicrograph (Hematoxylin and eosin (H&E), 100×) showing multiple epithelioid histiocytic granuloma (MEG, blue arrow) and Langhans giant cells (LGC, black arrows),; *lower middle*: photomicrograph showing granuloma (G, black arrows) with caseation necrosis (CN, blue arrow), H&E, 40×; *lower right:* intestinal tuberculosis, photomicrograph (H&E, 40×) showing illeal mucosa (IM, blue arrow) having epithelioid histiocytic granuloma (EHG, green arrow), Langhans giant cells (LGC, black arrow) and chronic inflammatory cell infiltrate (CI, yellow arrow).

During the study, 246 case-control subjects were recruited at the outpatient clinic of Rajan Babu Institute of Pulmonary Medicine and Tuberculosis hospital, Delhi, India. The detailed study methodology is depicted in Fig. 2. A total of 129 confirmed “TB patients” included 62 PTB and 67 EPTB cases. The “control” group (*n* = 41) included unrelated healthy individuals with no symptoms of any infection or previous history of TB (*n* = 22), household contacts who were exposed to active TB patients (*n* = 6), hospital controls who visited the hospital but TB diagnosis was ruled out (*n* = 13). Median age (in years) of PTB, EPTB cases and controls were 29.5 (Interquartile range (IQR) 21.75–41.25), 25 (IQR 18–34) and 26 (IQR 24–29), respectively. We also recruited two different categories of patients: 1) PTB and EPTB treatment-complete patients (TC) who had completed the standard anti-tubercular treatment (ATT) regimen (*n* = 39), and 2) non-TB respiratory patients diagnosed with asthma and chronic obstructive pulmonary disease (*n* = 19). This group was tested to look for efficacy of stool PCR in TC cases and to check for false positives due to any unrelated respiratory condition. Demographic and clinical characteristics of the TB patients are given in Table 1 and Supplementary Table S1.

**Table 1:** Demographic and clinical characteristics of TB patients.

Characteristics	PTB (n = 62)	EPTB (n = 67)	TC (n = 39)
**Age**			
[IQR: Median (range) in years]	29.5 (21.75–41.25)	25 (18–34)	23 (17–30)
0–14	03	07	06
15–45	46	50	30
>45	13	10	03
Male/Female	47/15	25/42	19/20
Relapse cases	15	15	NA
History of TB contact	05	10	04
Any comorbidity	04	03	02
**TB category**	**Number**	**IS6110 PCR**	**Sensitivity (95%CI)**
**Pulmonary:**			
Smear positive	36	35	97.22 (85.47–99.93)
Smear negative	26	20	76.92 (56.35–91.03)
**Extra-pulmonary:**			
Lymph node	34	25	73.53 (55.64–87.12)
*Cervical lymphadenopathy*	27	21	
*Mediastinal*	02	02	
*Others*	05	02	
Abdominal	15	10	66.67 (38.38–88.18)
*Mesenteric*	06	06	
*Intestinal*	02	02	
*Ascites*	07	02	
Pleural Effusion	16	09	56.25 (29.88–80.25)
Ocular	01	01	100 (2.50–100.00)
Uterus	01	01	100 (2.50–100.00)

PTB: Pulmonary tuberculosis, IQR: Interquartile range

EPTB: Extrapulmonary tuberculosis, CI: Confidence Interval

TC: Treatment complete.

### **Diagnostic performance and utility of stool PCR assay for detection of*M. tuberculosis*infection**

To ascertain reproducibility of stool PCR assay (Supplementary Figure S1), the study results are based on assay performed a minimum of two times for each stool sample for all subjects. Against the composite reference standard, the clinical sensitivity of stool PCR for PTB (*n* = 62) and EPTB (*n* = 67) groups were 88.71 [confidence interval, (CI), 78.11–95.34] and 68.66 [CI, 56.16–79.44], respectively (Table 2). Among the PTB group, sensitivity was higher among smear-positive cases [97.22 (CI, 85.47–99.93)] versus smear-negative cases [76.92 (CI, 56.35–91.03)]. Stool PCR testing for different EPTB groups showed maximum sensitivity for lymph node TB, 73.53 (CI, 55.64–87.12) followed by abdominal TB, 66.67 (CI, 38.38–88.18) and pleural effusion, 56.25 (CI, 29.88–80.25). A single case each of ocular and genital TB was reported in the cohort that also tested positive by stool PCR (Table 1). Stool PCR presented a specificity of 95.12 (CI, 83.47–99.40). All subjects in the non-TB respiratory patient group (19/19), unrelated healthy control (22/22) and hospital control (13/13) group tested negative by stool PCR, while 2/6 household contact controls and 7/39 TC cases were positive by stool PCR.

**Table 2:** Diagnostic accuracy of stool PCR and stool Xpert MTB/RIF for microbiologically/clinically confirmed TB patients.

Molecular test	^a^Reference standard (Confirmed TB cases)	
	PTB (n = 62)	EPTB (n = 67)
**Stool IS6110 PCR**		
^b^Sensitivity (%; 95% CI)	55/62; 88.71 (78.11–95.34)	46/67; 68.66 (56.16–79.44)
^c^Specificity (%; 95% CI)	2/41; 95.12 (83.47–99.40)	2/41; 95.12 (83.47–99.40)
^d^PPV (%; 95% CI)	96.49 (87.65–99.07)	95.83 (85.50–98.90)
^e^NPV (%; 95% CI)	84.78 (73.43–91.83)	65.00 (56.41–72.71)
^f^Accuracy (%; 95% CI)	91.26 (84.06–95.93)	78.70 (69.78–86.00)
**Stool GeneXpert**		
^b^Sensitivity (%; 95% CI)	11/15; 73.33 (44.90–92.21)	2/12; 16.67 (2.09–48.41)
^c^Specificity (%; 95% CI)	0/9; 100.00 (66.37–100.00)	0/9; 100.00 (66.37–100.00)
^d^PPV (%; 95% CI)	100.00	100.00
^e^NPV (%; 95% CI)	69.23 (49.29–83.89)	47.37 (41.13–53.69)
^f^Accuracy (%; 95% CI)	83.33 (62.62–95.26)	52.38 (29.78–74.29)

^a^Reference standard: Clinician’s decision to treat TB

^b^Sensitivity: True positives (TP)/[TP + False negatives (FN)]

^c^Specificity: True negatives (TN)/[TN + False positives (FP)]

^d^PPV: Positive predictive value- TP/[TP + FP]

^e^NPV: Negative predictive value- TN/[TN + FN]

^f^Accuracy: TP + TN/[TP + FP + TN + FN].

**Figure 3:**
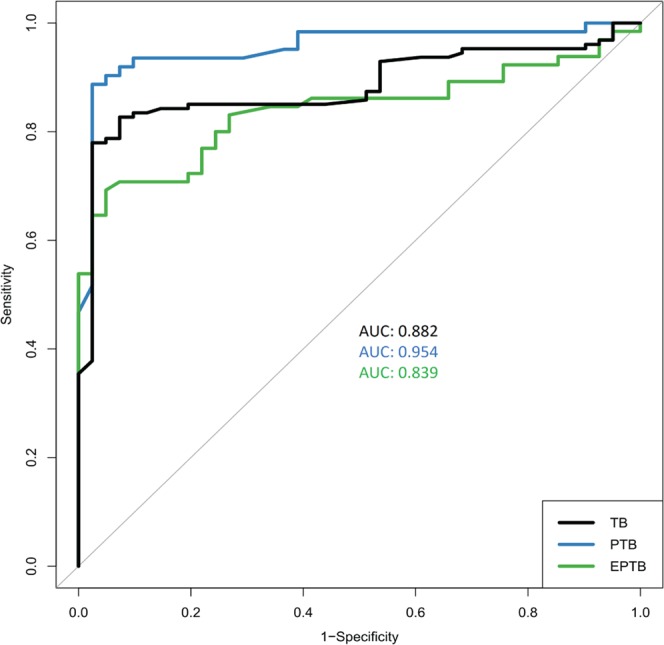
ROC curve of *M. tuberculosis* DNA detection by Stool PCR. Area under the ROC curve (AUC) values of PTB, EPTB and combined TB (PTB + EPTB) are shown.

A highly significant positive association of *Mtb* detection with stool PCR was obtained in both manifestations of TB (*p* < 0.001). While male sex was significantly associated with only PTB, inclusion of both sex and age as predictors in the logistic regression model helped in achieving a better fit (Supplementary Table S2). Stool PCR had a high accuracy rate of *Mtb* detection, as revealed by the receiver operating characteristic (ROC) curve [area under the ROC curve (AUC): 0.882; CI, 0.829–0.935]. Upon comparison, PCR more accurately classified PTB cases as compared to EPTB, as reflected by an increased AUC [0.954 (CI, 0.913–0.996) for PTB versus 0.839 (CI, 0.763–0.916) for EPTB] (Fig. 3). The Youden index for the ROC curve, which can be used as the optimal diagnostic cutoff value for clinical specimens, was 0.965 for TB detection.

Logistic regression analysis also revealed significant association of stool PCR positivity with occurrence of both PTB and positive acid-fast bacilli smear status (Supplementary Table S3) and presence of EPTB at various sites (Supplementary Table S4). Notably, age and gender did not show significant association with performance of PCR in TB (Supplementary Tables S3 and S4).

### Diagnostic usefulness of stool with Xpert assay

To test the adaptability of stool PCR in a clinical set-up, Xpert testing was performed in a pilot run of 36 samples. Stool Xpert yielded positive results in 11/15 (73%) PTB (7 smear-positive and 4 smear-negative) and 2/12 (17%) EPTB cases, while specificity was 100% (Table 2). Stool PCR was earlier positive for 23/27 of these cases. The two stool Xpert positive EPTB cases were of lymphadenopathy and pleural effusion; and the lymphadenopathy case also tested positive for rifampicin resistance. Upon comparison of the Ct values from *Mtb* positive stool samples obtained by Xpert analysis, Ct values associated with EPTB cases were much higher than those from PTB cases (Supplementary Figure S2), highlighting the low detection ability of Xpert in paucibacillary samples.

## Discussion

This study supports the utility of stool as a viable non-invasive, alternate sample for TB diagnosis. Our results show that molecular testing of stool samples can detect both smear-positive and smear-negative PTB patients, with high accuracy (91.3%, Table 2). It also demonstrates, for the first time, the diagnostic accuracy (78.7%) of stool as a sample for PCR detection of diverse EPTB forms. The specificity of stool molecular test was also excellent as only 2 household contacts out of 41 controls (95.12%) tested positive by stool PCR and all stool samples from healthy individuals were negative (100%) by Xpert assay. We were unable to test these PCR-positive household contacts by standard investigation. Nonetheless, the results strongly indicate that examination of stool specimens may facilitate routine screening of high-risk cases, an important aspect in endemic regions. Meanwhile, each of the stool samples from the non-TB respiratory patient group was negative for *Mtb* (specificity 100%), which shows the low probability of false-positive results. ROC curve analysis, a widely applied statistical tool to determine reliability of the diagnostic method, also provided evidence for clinical utility of stool PCR in *Mtb* detection (AUC: 0.882) in varied TB cases.

Bacilli DNA detection methods are generally not considered amendable for monitoring treatment response since a positive result may not imply the viability of the pathogen. However, the PCR results of TC patients show high concurrence with clinical trend, as only 7/39 patients tested positive. Three (3EPTB) out of these 7 TC cases (1PTB/6EPTB) were advised extended ATT regimen on basis of their existing clinical symptoms. Although we could not collect samples from patients undergoing ATT, an earlier study had quantified mycobacterial load at 2 months of ATT by a stool-based quantitative PCR assay in PTB patients^17^. To our knowledge, these are the only studies exploring the potential of stool-based assays in ATT monitoring and more such studies are needed, since monitoring is especially difficult in paucibacillary TB patients.

One of the major findings of our study was the ability of PCR to detect *Mtb* DNA in stool samples from EPTB patients displaying varied forms, such as lymph node TB, abdominal TB, pleural effusion, genital TB and ocular TB. While an earlier study had shown extrapulmonary *Mtb* detection by stool Xpert of a single patient sample, but the nature of infection was unknown^18^. Fecal PCR has also been used earlier to identify intestinal TB^19^, but our study reports sensitivity of stool PCR in various abdominal TB categories, including intestinal TB, mesenteric lymph nodes and ascites. Earlier reports on Xpert with direct site specimens of EPTB patients have shown highest test sensitivity with lymph nodes and lowest sensitivities in pleural fluid and ascites^20–22^. Interestingly, our stool PCR results also show similar trend, possibly reflecting the total body burden of mycobacterial infection in different forms. The exact mechanism of mycobacterial DNA shedding in stool still needs to be explored but emerging evidence suggests bi-directional exchange of metabolites, immune cells and bacterial fragments between gut and lung by use of systemic circulation^23^. However, these results warrant deeper insights, as the mechanistic basis of EPTB remains unsolved. A major challenge in the diagnosis of EPTB is the non-specific and diverse clinical presentation that delays confirmation. So, detection of mycobacterial DNA in stool of EPTB patients may be potentially useful as a rule-in test.

While Xpert on stool has still not been included in recommendations by the WHO, emerging evidence on stool Xpert for pediatric PTB, strongly advocates its usefulness. A recent meta-analysis of stool Xpert for children showed pooled sensitivity and specificity of 67% and 99%, respectively^12^. Although data on molecular testing of stool for adults is limited, high sensitivity and specificity is reported with stool PCR and stool Xpert in PTB patients^8,13,14^. In a run with limited samples, our results on stool Xpert also showed high accuracy (83.3%) for PTB but tested positive for only 2/12 EPTB patient samples. This discordance in results from both methods used in our study may be attributed to the difference in copy numbers of target genes (*IS6110* and *rpoB*), which may have contributed to difference in sensitivities^24^. It is pertinent to note that the use of new-generation Xpert-Ultra assay has resulted in increased sensitivity of mycobacterial DNA detection in extrapulmonary samples, including stool^20^.

Although the preliminary results are promising, the major limitations of this study include the small number of samples in Xpert run. Nevertheless, our results do indicate that stool Xpert is able to detect TB cases with high specificity and high sensitivity in PTB, albeit with low sensitivity in EPTB cases. High detection rate of EPTB cases in stool PCR emphasizes the diagnostic potential and testing with Xpert Ultra would provide better insights. Second, lack of any confirmed drug-resistant and human immunodeficiency virus-positive cases in the study hindered our ability to make any judgment on test sensitivity under such conditions. One of the drawbacks with the current diagnostic algorithm is lack of routine use of CT scan of patients, and in its absence, one cannot completely rule out pulmonary involvement in all the EPTB cases, since X-ray is unable to detect remote lesions.

Studies with larger sample size would help to better elucidate the diagnostic accuracy of Xpert/Xpert-Ultra in stool samples from PTB and EPTB patients. More work on TB testing with stool samples would lead to more optimized methods for processing stool^15,16^, which can have positive implications in diagnosis of paucibacillary cases. Timely screening and rapid detection of *Mtb* in clinical specimens is crucial for control of TB. Molecular testing of stool may facilitate TB diagnosis in subjects who are unable to produce sputum, or have EPTB and or in screening of high-risk groups.

## Methods

### Study design and participants

Subjects were recruited for the study between August 2017 and March 2018 at the outpatient clinic of Rajan Babu Institute of Pulmonary Medicine and Tuberculosis hospital, Delhi, India. All patients were prospectively recruited based on presumptive TB features. The patients’ inclusion (*n* = 129) for this study was following confirmation of PTB and EPTB by the clinician. Stool sample was collected from patient group prior to treatment or as soon as treatment commenced (within 2–3 days). The control subjects (*n* = 41) included unrelated healthy individuals: *n* = 22, household contacts: *n* = 6, and hospital controls: *n* = 13. We also collected stool samples from PTB and EPTB patients who had completed the ATT (*n* = 39), and patients with other unrelated respiratory diseases (*n* = 19). Each subject was provided with a sterile container for collection of the stool sample. The samples were frozen at −80 °C in aliquots on procurement and processed within 72 hours for stool PCR. All participants gave written informed consent and for those patients under the age of 18 years, informed consent was obtained from a parent/guardian. The Institutional ethics committee at Rajan Babu Institute of Pulmonary Medicine and Tuberculosis approved the recruitment criteria and study protocol (2027/RBIPMT/2017). All methods were performed in accordance with the principles laid by the Indian Council of Medical Research.

### Stool DNA extraction and IS6110-PCR

DNA was extracted from all stool samples (200 mg) using the QIAamp DNA stool mini kit (Qiagen, USA), according to manufacturer’s instructions, with slight modifications, as previously described^25^. DNA concentration and quality were estimated using a Nanodrop instrument (Thermo Scientific) and agarose gel electrophoresis. The purified DNA was diluted to a final concentration of 100 ng/μl with nuclease free water (Thermo Scientific). PCR amplification of the insertion segment IS6110 was performed on each sample with two independent sets of primers, to amplify fragments of 123 bp^26^, and 343 bp (F-5′TCGGACCACCAGCACCTAAC3′ & R-5′GTCTCGGCTAGTGCATTGTC3′), respectively. A PCR run with universal 16S primer set: (P3F- 5′CGATCCCTAGCTGGTCTGAG3′, P3R- 5′GTTAGCCGGTGCTTCTTCTG3′) was also done to rule out any PCR inhibitors. All the primers were supplied by Sigma Aldrich. DNA amplification was performed in a thermocycler (Bio-Rad, T100^TM^), in a final volume of 20 μl containing 5X PCR buffer, 2.5 mM dNTPs, 20 pmoles of each primer, 10%DMSO, 1U KOD DNA polymerase (Sigma Aldrich) and 100–200 ng of template DNA. Cycling conditions while using 123bp-product primers were: initial denaturation at 95 °C for 5 min, followed by 35 cycles of denaturation at 95 °C for 20 s, annealing at 66 °C for 10 s, extension at 72 °C for 5 s, and a final extension at 72 °C for 10 s. For the 343 bp IS6110 gene fragment amplification, a minor modification of annealing at 66 °C for 20 s was included. *M. tuberculosis* H37Rv genomic DNA as positive control and mastermix without DNA sample as negative control were included in each PCR run.

### Xpert MTB/RIF assay

The Xpert assay was performed according to the manufacturer’s instructions with slight modifications at Lok Nayak Hospital, Delhi, India. Briefly, 200 mg thawed stool samples were mixed with 2.4 ml phosphate buffered saline, vortexed thrice and incubated at room temperature for 20 min. One ml aliquot of supernatant was centrifuged at 3200 g for 15 min. The pellet was resuspended in 1 ml phosphate buffered saline and 2 ml Xpert sample-reagent was added to the specimen. This mixture was immediately transferred to the Xpert cartridge and inserted into the GeneXpert instrument (Cepheid, CA).

### Statistical analysis

Clinical sensitivity, specificity, positive predictive values and negative predictive values were calculated for stool PCR and stool Xpert at 95% CI, using microbiologically/clinically confirmed TB as the reference standard. Statistical analysis was performed between TB patients (*n* = 129) and controls (*n* = 41). To assess the diagnostic value of stool-PCR in TB detection, we made a logistic regression model for predicting TB using stool PCR, age and sex as predictors. ROC curve analysis was generated, with calculation of AUC for TB detection. The optimal diagnostic cutoff value was determined by calculating the Youden index of the ROC curve. The ROC curve analysis was performed using pROC package of R (v3.4.4)^27^. Associations between stool PCR positivity and factors such as form of TB (PTB/EPTB), age, sex were calculated as odds ratios with their 95% CI by logistic regression. A multinomial logistic regression model was built for EPTB to include the multiple categories for disease location. Logistic regression models excluded the single cases of ocular and genital TB in patients and were built by generalized linear model: glm() function of R.

## Supplementary information

Supplementary Information.

Supplementary Information 2.
