# Supplementary material for: Diagnostic performance of non-invasive, stool-based molecular assays in patients with paucibacillary tuberculosis
Source: Sci Rep. 2020 Apr 28;10:7102. doi: 10.1038/s41598-020-63901-z (PMC7188812; doi:10.1038/s41598-020-63901-z)
Supplement: Supplementary file 1 — Supplementary Information. [file 41598_2020_63901_MOESM1_ESM.docx]

**Supplementary Information**

**Diagnostic performance of non-invasive, stool-based molecular assays in patients with paucibacillary tuberculosis**

Mohita Gaur^1^, Anoop Singh^1^, Vishal Sharma^1^, Gayatri Tandon^1^, Ankur Bothra^1^, Aarushi Vasudeva^1^, Shreeya Kedia^1^, Ashwani Khanna^2^, Vishal Khanna^3^, Sheelu Lohiya^3^, Mandira Varma-Basil^4^, Anil Chaudhry^5^, Richa Misra*^1,6^, Yogendra Singh*^1^

^1^Department of Zoology, University of Delhi, Delhi.

^2^State TB Officer & In-Charge, Chest Clinic, Lok Nayak Hospital, Delhi

^3^Chest Clinic, Lok Nayak Hospital, Delhi

^4^Vallabhbhai Patel Chest Institute, University of Delhi, Delhi

^5^Rajan Babu Institute of Pulmonary Medicine and Tuberculosis, Kingsway Camp, Delhi

^6^Sri Venkateswara College, University of Delhi, Delhi

**Supplementary Table S2: Logistic regression model for TB**

| **B(SE)^a^ 95% CI for OR** | | | | |
| --- | --- | --- | --- | --- |
| Lower OR Upper | | | | |
| **TB** |  |  |  |  |
| b_0_ (constant) | -1.02 (0.55) |  |  |  |
| Stool PCR | 4.26 (0.76)*** | 19.72 | 71.11 | 459.29 |
| Sex (male) | 0.35 (0.48) | 0.55 | 1.42 | 3.70 |
| Age | 0.02 (0.01) | 0.99 | 1.02 | 1.05 |
|  | | | | |
| **PTB** |  |  |  |  |
| b_0_ (constant) | -4.01(1.14) |  |  |  |
| Stool PCR | 5.41 (0.98)*** | 42.74 | 223.15 | 2267.10 |
| Sex (male) | 1.79 (0.86)* | 1.26 | 6.00 | 43.31 |
| Age | 0.04 (0.02) | 0.99 | 1.04 | 1.09 |
|  | | | | |
| **EPTB** |  |  |  |  |
| b_0_ (constant) | -1.05 (0.60) |  |  |  |
| Stool PCR | 3.84 (0.79)*** | 12.18 | 46.45 | 310.13 |
| Sex (male) | -0.20 (0.54) | 0.28 | 0.82 | 2.33 |
| Age | 0.02 (0.02) | 0.98 | 1.02 | 1.05 |

^a^B(SE), Regression coefficient (standard error) TB, Tuberculosis (PTB+EPTB)

CI, Confidence Interval, OR, Odds ratio PTB, Pulmonary tuberculosis

b_0_, Y-intercept EPTB, Extrapulmonary tuberculosis

*p* value < 0.05 (*); <0.001 (***)

**Supplementary Table S3: Logistic regression analysis exploring factors associated with stool PCR positivity in PTB cases**

| **Variable OR 95% CI *p*-value** |
| --- |
| **Sex**  Female Reference  Male 1.10 0.14-6.03 0.9 |
|  |
| **TB occurrence**  No TB Reference  PTB 84.64 15.24-870.36 <0.001*** |
|  |
| **Age**  0.96 0.92-1.01 0.1 |
|  |
| **AFB Status**  Negative Reference  Positive 10.95 1.64-218.82 <0.05* |

OR, odds ratio ‘Reference’ refers to the base or reference category used for the regression analyses.

CI, confidence interval

AFB, acid-fast bacilli

**Supplementary Table S4**: **Multinomial logistic regression analysis exploring factors associated with stool PCR positivity in EPTB cases**

| **Variable OR 95% CI *p*-value** |
| --- |
| **Sex**  Female Reference  Male 1.88 0.62-5.70 0.26 |
|  |
| **Site**  No TB Reference  Lymph node 57.37 11.05-297.78 <0.001***  Abdominal 44.47 7.07-279.54 <0.001***  Pleural effusion 29.85 4.96-179.87 <0.001*** |
|  |
| **Age** 0.97 0.94-1.00 0.08 |

OR, odds ratio ‘Reference’ refers to the base or reference category used for the regression analyses.

CI, confidence interval


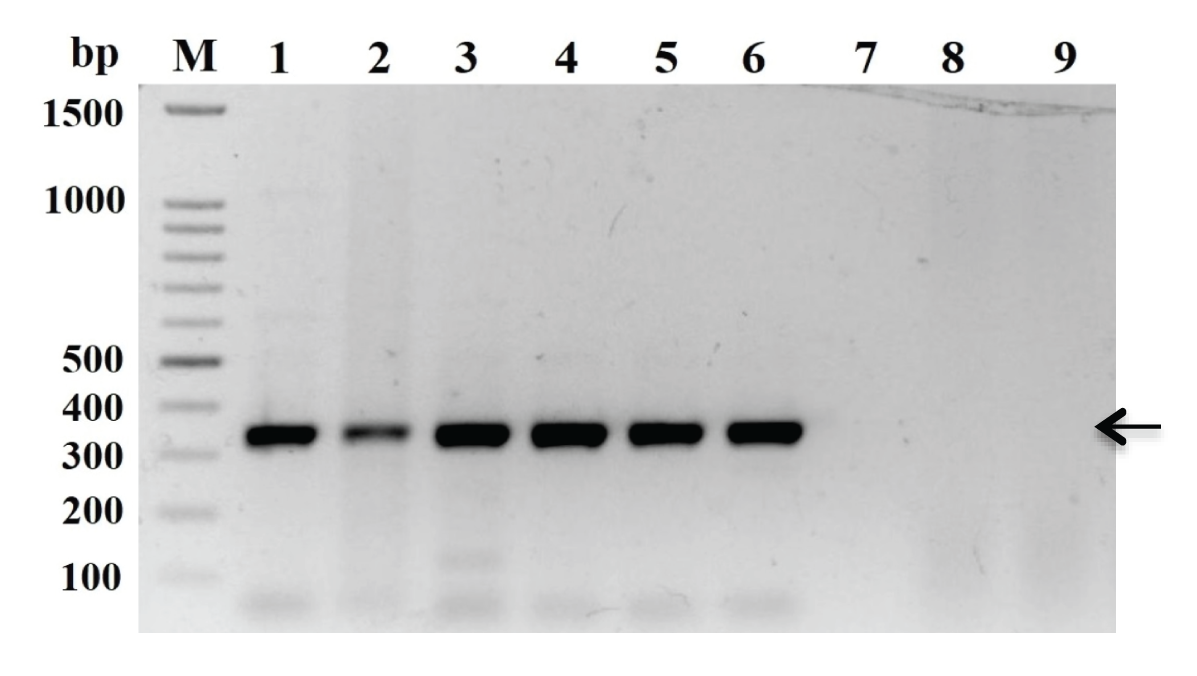


**Supplementary Figure S1**: **PCR amplification of the 343 bp fragment of *IS6110* gene of *M. tuberculosis* complex**. Agarose gel electrophoresis (1.2%) of the 343 bp amplicon from stool samples, *lane M*, 100 bp DNA ladder (Gene Direx), *lane 1,* H37Rv genomic DNA (positive control), *lanes 2-4*, extrapulmonary patient stool samples, *lanes* 5-6, pulmonary patient stool samples, *lane 7*, water, *lanes 8-9*, control stool samples


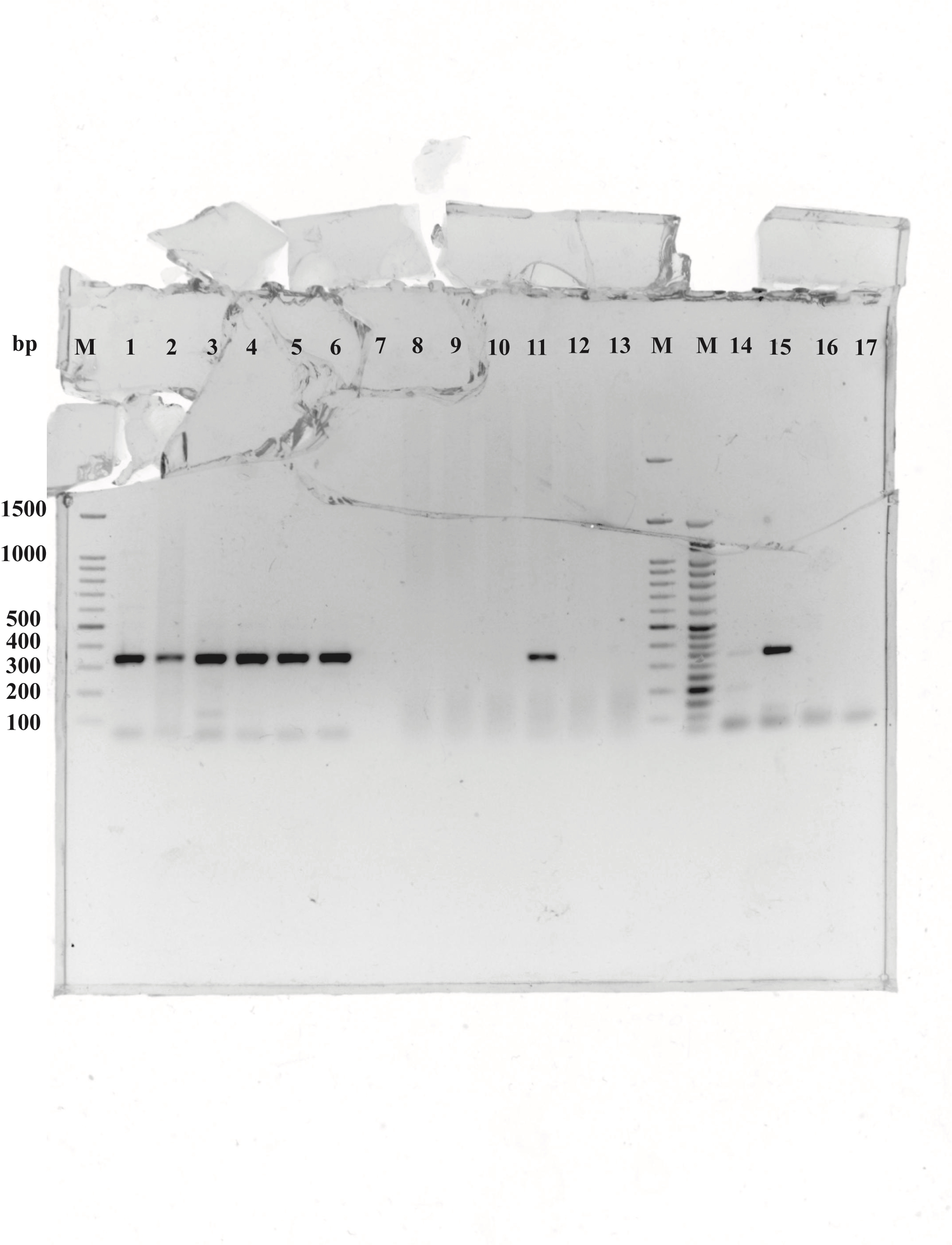


**Original file for Supplementary Figure S1**: **PCR amplification of the 343 bp fragment of *IS6110* gene of *M. tuberculosis* complex**. Agarose gel electrophoresis (1.2%) of the 343 bp amplicon from stool samples, *lane M*, 100 bp DNA ladder (Gene Direx), *lane 1,* H37Rv genomic DNA (positive control), *lanes 2-4*, extrapulmonary patient stool samples, *lanes* 5-6, pulmonary patient stool samples, *lane 7*, water, *lanes 8-9*, control stool samples

Lane 10, water, lane 11, BCG genomic DNA, lane 12-13, control samples, lane M, DNA Markers, lane 14-17 Gradient PCR for 343 bp amplicon from H37Rv genomic DNA


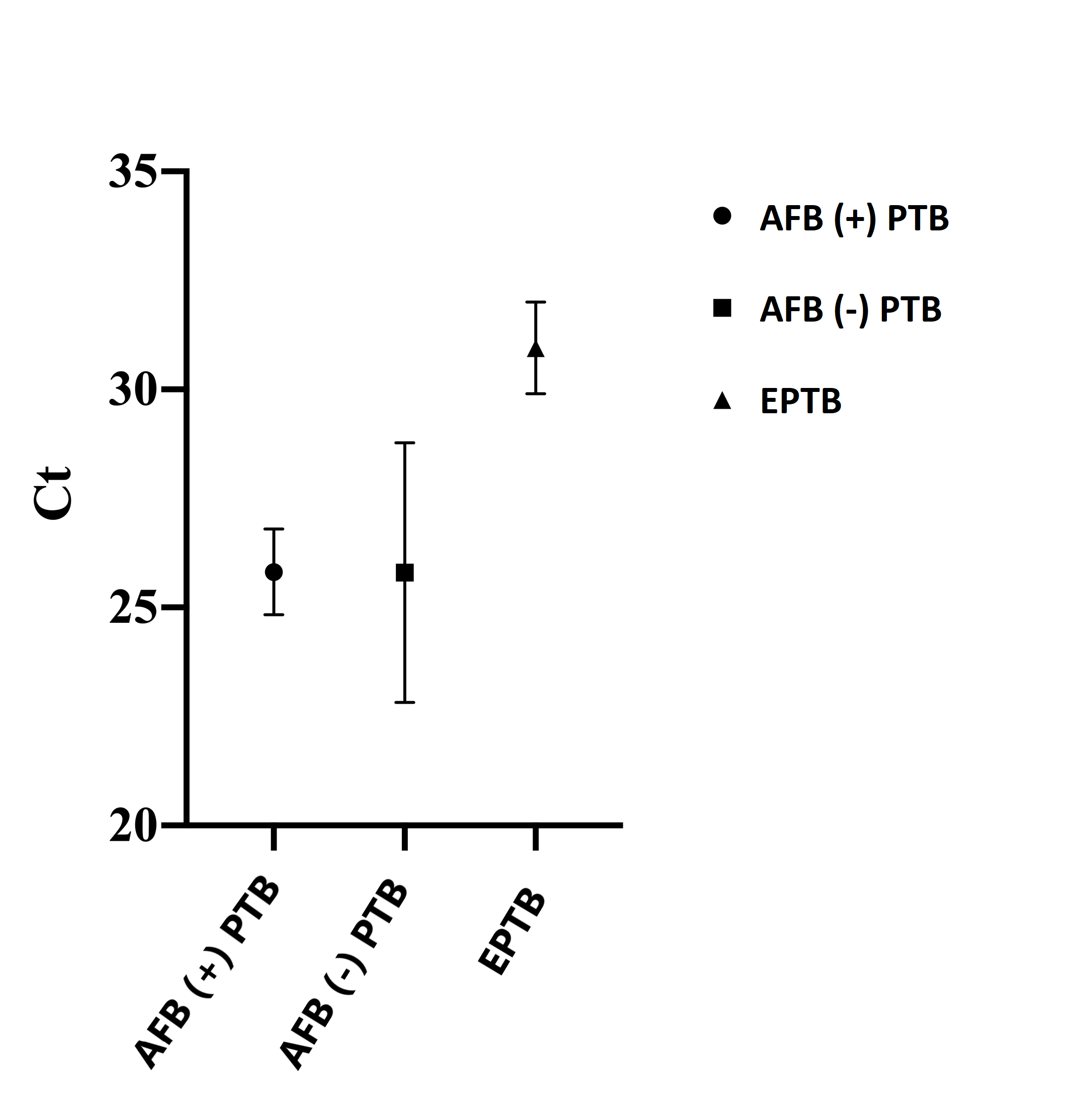


**Supplementary Figure S2**: **Relationship between Xpert MTB/RIF-generated cycle threshold (Ct) values and TB patient groups.** Plot depicts the mean and standard deviation of Ct values obtained from stool samples of AFB (+) PTB, AFB (-) PTB and EPTB patients, tested on Xpert MTB/RIF. Minimum Ct value of probe was taken for calculation of mean for each subject. The Ct of the first positive probe was used to identify the minimum Ct value of each assay.
